# Spinal cord stimulation for postural abnormalities in Parkinson’s disease: 1-year prospective pilot study

**DOI:** 10.1186/s12883-024-03673-5

**Published:** 2024-05-21

**Authors:** Liche Zhou, Fangzheng Chen, Yixin Pan, Linbin Wang, Lu Xu, Peng Huang, Lijun Wang, Ningdi Luo, Puyu Li, Dianyou Li, Jun Liu

**Affiliations:** 1 Department of Neurology, Institute of Neurology, Ruijin Hospital, Shanghai Jiao Tong University School of Medicine, Shanghai, 200025 China; 2 Department of Neurosurgery, Ruijin Hospital, Shanghai Jiao Tong University School of Medicine, Shanghai, 200025 China; 3 Institute of Science and Technology for Brain-Inspired Intelligence (ISTBI), Fudan University, Shanghai, 200433 China; 4 Neurovascular Center, Changhai Hospital, Naval Medical University, Shanghai, 200433 China

**Keywords:** Parkinson’s disease, Spinal cord stimulation, Postural abnormalities

## Abstract

**Background:**

Postural abnormalities (PA) are common in the advanced stages of Parkinson’s disease (PD), but effective therapies are lacking. A few studies suggested that spinal cord stimulation (SCS) could be a potential therapy whereas its effect is still uncertain. We aimed to investigate whether SCS had potential for benefiting PD patients with PA.

**Methods:**

T8-12 SCS was operated on six PD patients with PA and all patients were followed for one year. Evaluations were made before and after SCS. Moreover, three patients were tested separately with SCS on-state and off-state to confirm the efficacy of SCS.

**Results:**

Improvements in lateral trunk flexion degree, anterior thoracolumbar flexion degree and motor function were found after SCS. The improvements diminished while SCS was turned off.

**Conclusions:**

Lower thoracic SCS may be effective for improving PA in PD patients, but further studies are needed to confirm this conclusion.

**Trial registration:**

Chinese Clinical Trial Registry, ChiCTR1900024326, Registered on 6th July 2019; https://www.chictr.org.cn/showproj.aspx?proj=40835.

**Supplementary Information:**

The online version contains supplementary material available at 10.1186/s12883-024-03673-5.

## Background

Parkinson’s disease (PD) is a common neurodegenerative disorder in which the main cause is basal ganglia dysfunction, resulting from degeneration of neurons in the dopaminergic nigrostriatal pathway [1]. Dopaminergic medication can usually ameliorate the patient’s symptoms in the early stages of PD, but its efficacy decreases with the disease progressing and is prone to some long-term motor side-effects. When the disease progresses to the advanced stage, patients develop specific symptoms which tend to be less responsive to dopaminergic therapy, such as axial symptoms [2]. And medications cannot maintain long-term efficacy [3]. So, exploring effective treatments for axial symptoms in PD is in utmost need and has been a hot topic in current PD clinical research.

Lately, some studies have explored the application of SCS in PD [4–9]. A case report found an antiparkinsonian effect of SCS in a patient implanted for lower limb neuropathic pain [9], and some studies reported SCS might benefit advanced PD patients’ motor and gait function [5, 7, 8]. However, a prospective trial had reported no clinically meaningful effect of SCS for PD [6]. Therefore, some valuable insights were provided, but the effect of SCS was still uncertain. A previous study of our team reported that PD patients with postural abnormalities (PA) featured a significant severity in motor dysfunction, decreased pelvic obliquity angle, and more doses of dopaminergic medications needed [10]. According to previous studies of SCS, we considered that SCS might be a supplementary therapy for these patients.

In the present study, we performed SCS surgery on spinal segments T8–12 of six PD patients with PA. All six patients completed evaluations and were followed for one year. Furthermore, three of them were tested separately with SCS on-state and off-state to confirm the efficacy of SCS. The aim of this study is to explore the application of SCS in treating PD patients with PA.

## Methods

### Patients

We performed the study at Ruijin Hospital, Shanghai Jiao Tong University School of Medicine from June 2019 to December 2021. This study was approved by the ethics committee of Ruijin Hospital, Shanghai Jiao Tong University School of Medicine (2019-62), and registered at Chinese Clinical Trial Registry (ChiCTR1900024326). The protocol of the current study fitted the guidelines of the Declaration of Helsinki and its later amendments. Written informed consents were obtained from participants. Our study adhered to CONSORT guidelines.

**Fig. 1:**
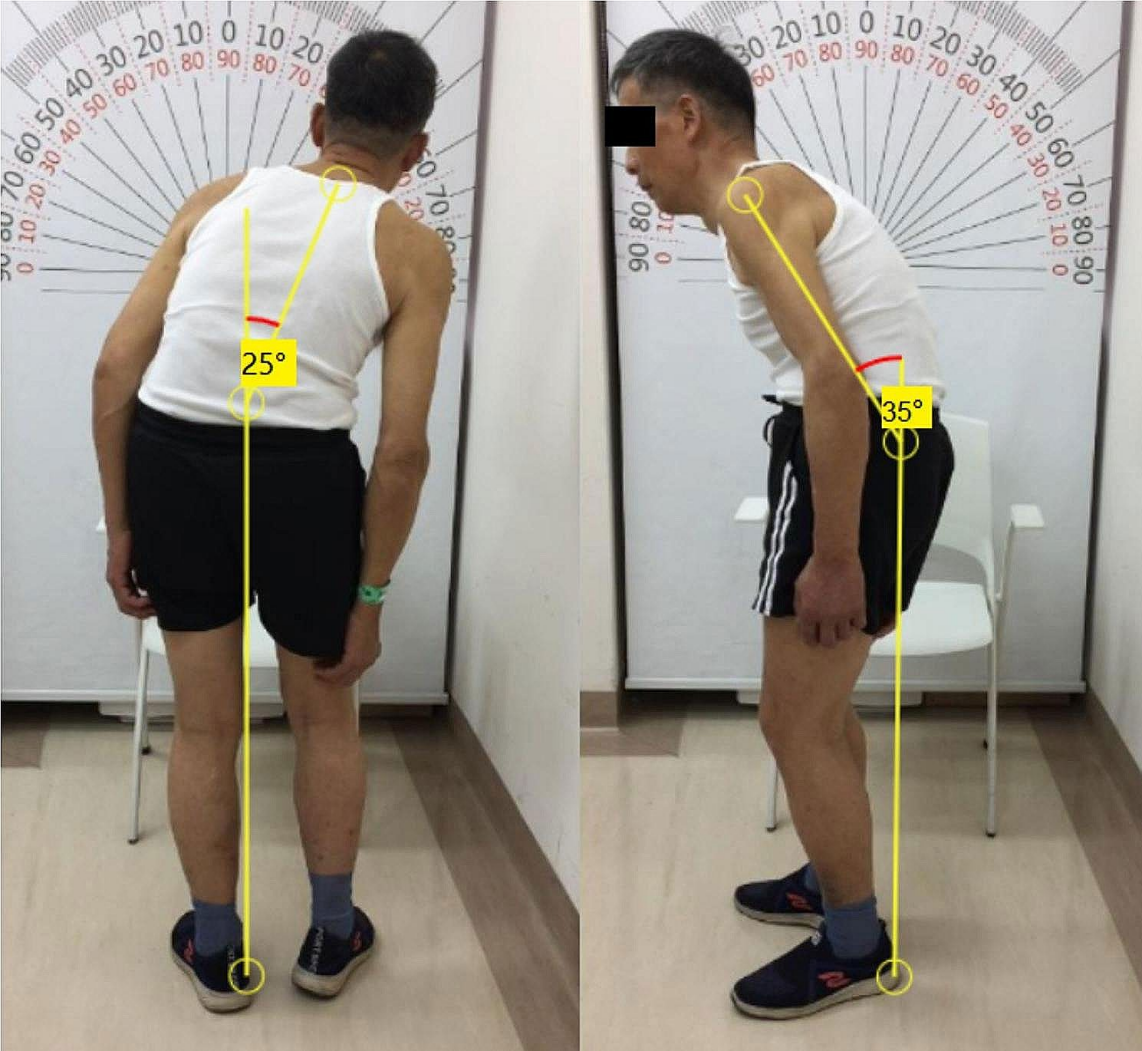
Measurement of the degree of lateral trunk flexion and anterior thoracolumbar flexion

PD patients actively seeking surgical treatment for postural disorders were recruited. PD was diagnosed according to the diagnostic criteria of the Movement Disorders Society (MDS) [11] by two experienced movement disorder specialists and patients were evaluated with the Movement Disorder Society Unified Parkinson’s Disease Rating Scale (MDS-UPDRS) [12] and the Hoehn-Yahr stage [13]. All participants met the following criteria: (a) age between 45 and 75 years, (b) no sign of dementia according to the Chinese version of the Mini-Mental State Examination (cMMSE) [14], (c) Hoehn-Yahr stage > 2, (d) PD patients with MDS-UPDRS Q 3.13-Posture scale > = 2 were recruited, and these patients’ postural abnormalities were minimally improved by levodopa in the “on” state, (e) no psychiatric disorders, (f) no history of other disorders involving the nervous system and musculoskeletal system, or of intracranial surgery or traumatic brain injury, (g) the reversibility during lying position had been verified in the patient. Patients were excluded at the baseline if they were unable to walk at least 5 m continuously without any assistance. The sagittal angle between a vertical line and a line connecting the trochanter with the edge of the acromion was evaluated to define anterior thoracolumbar flexion, and the coronal angle between a vertical line and a line passing through the C7 and L4 vertebrae was used to define lateral trunk flexion [15–17] (Fig. 1). We used web-based tools to measure the angles of lateral trunk flexion and anterior thoracolumbar flexion (http://www.neurologie.unikiel.de/de/axial-posturale-stoerungen/camptoapp) according to the recommendation of the consensus [18, 19].

Evaluations were made before the SCS surgery and 3, 6, 12 months after the SCS surgery. Three of all six patients were evaluated separately with SCS in the on- and off-states during follow-up (6 or 12 months). Evaluations of SCS off-state were made with the SCS system turned off for at least 24 h. Examiners were blinded during the on/off comparison. The patients were also evaluated using the Scale for Outcomes in PD-Autonomic (SCOPA-AUT) [20], 39-item quality of life questionnaire for Parkinson’s disease (PDQ-39) [21] and Wexner constipation score (WCS) [22].

### SCS procedure

Two cylindrical percutaneous electrodes with eight contacts per lead (Model 3777, Medtronic) were implanted in the medial epidural space at spinal segments T8–12 under local anesthesia, with the electrode positioned to produce paresthesia fully covering the lower trunk and lower extremities including both legs and feet.

On the second day after lead implantation, the lead contacts were activated with an external stimulator, stimulating at 40–60 Hz to produce paresthesia covering the lower trunk and lower extremities. If the clinical response (A sign of postural improvement presented and no unwanted effects occurred) in the test phase (less than 4 weeks) was satisfactory, patients underwent second-stage surgery to implant a paddle lead with three columns of contacts (5–6–5 Model 39,565; Medtronic) into the epidural space of the thoracic spine (T8–T12) by partial laminectomy, under X -ray magnification. The lead was then connected with the pulse generator (Model 37,714, Medtronic) in the subcutaneous pocket at the abdomen.

One month after the surgical lead implantation, positional adaptation function was applied. Paresthesia was only clearly sensed when the patients were sitting or walking and was nearly imperceptible when they were lying down.

### Statistical analysis

Statistical analysis was performed using SPSS V.22.0. The clinical data were used to calculate the mean and rate of change at each follow-up time. We analyzed measures of participants using paired Wilcoxon’s test depending on the type and distribution of dependent variables. *P* < 0.05 was considered statistically significant for all analyses. All tests were two-tailed.

## Results

**Fig. 2:**
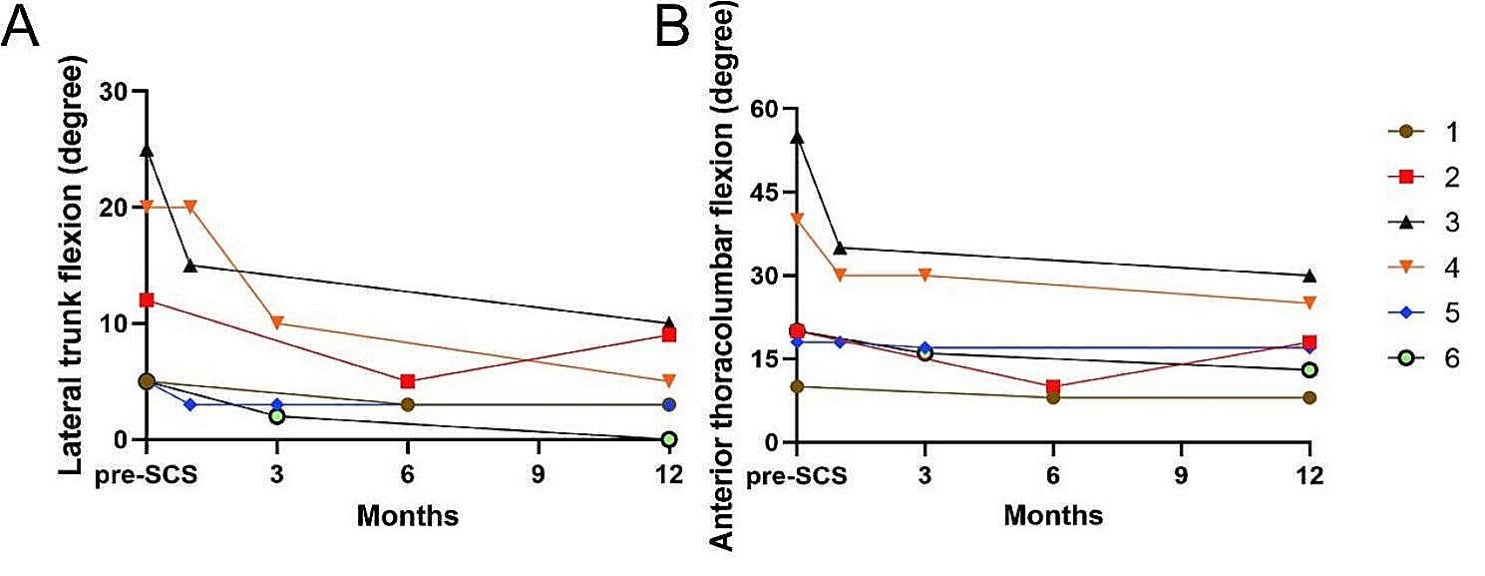
Individual outcomes of axial parameters before SCS and follow-up after surgery during ongoing stimulation. **(A)** Changes in degree of lateral trunk flexion. **(B)** Changes in degree of anterior thoracolumnbar flexion. Each line represents one patient

**Fig. 3:**
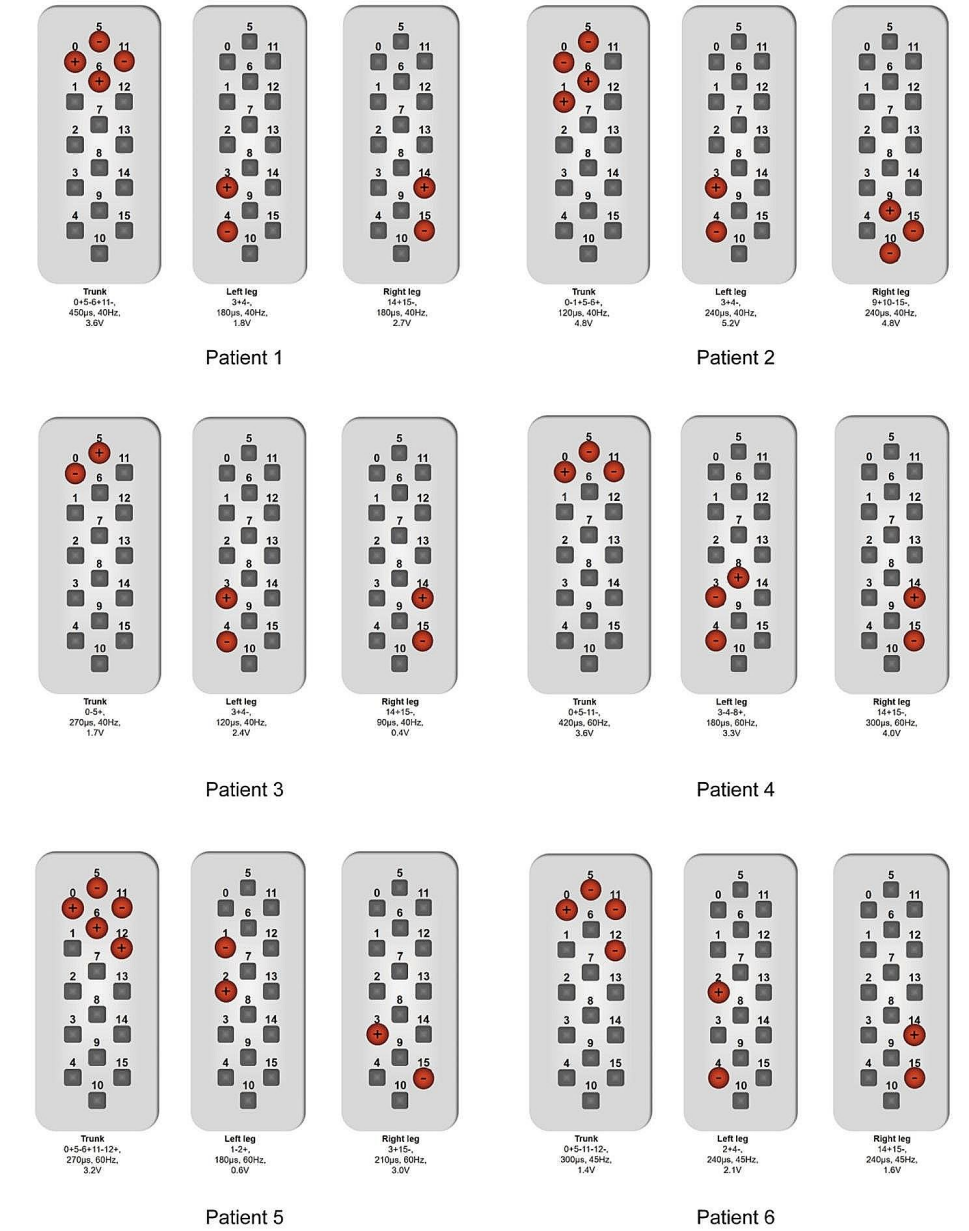
The settings for SCS of each patient. All of the above settings were recorded one month after the surgical lead implantation. SCS: Spinal Cord Stimulation

The demographic and clinical characteristics of all participants are shown in Table 1. The assessments were recorded on patients’ “medication-on” status after regular medications with SCS on-state. Evaluations made before the SCS surgery are shown in Table 2. During the one-year follow-up, no extra antiparkinsonian drugs were needed in all participants. There were significant differences in the degree of lateral trunk flexion (*p* < 0.027) and anterior thoracolumbar flexion (*p* < 0.028) before SCS surgery and at the one-year follow-up. There was no significant difference in the MDS-UPDRS III score (*p* > 0.05) before SCS surgery and at the one-year follow-up. After SCS, the mean lateral trunk flexion degree was improved by 58%, mean anterior thoracolumbar flexion degree was improved by 32% and the mean MDS-UPDRS-III score was improved by 9% (Table 1; Fig. 2). Scores of specific items in MDS-UPDRS III for patients before SCS surgery and at the one-year follow-up were presented in Supplementary Table 1. In majority of patients, the scores of MDS-UPDRS Q 3.3 (d/e) – Rigidity (Right Lower Extreme/Left Lower Extremity), MDS-UPDRS Q3.4 - Finger tapping and MDS-UPDRS Q 3.7 - Toe tapping are lower at the one-year follow-up. In all, the motor function of lower extremities was improved in most patients. Settings for SCS of each patient are shown in Fig. 3.

**Table 1:** Participants’ demographics and clinical measurements at pre-SCS and after SCS implantation

Patient ID		1	2	3	4	5	6	Mean ± SD	Improvement rate
Pre-SCS/ Last follow-up after SCS implantation	Age (years)	72	72	68	71	64	72	70 ± 3	/
	Gender	Female	Male	Male	Male	Female	Male	/	/
	Disease duration (years)	8.1	4.0	12.4	5.0	17.1	8.0	9.1 ± 4.9	/
	PA duration (years)	3.5	0.5	7.0	2.0	4.0	6.0	3.8 ± 2.4	/
	Hoehn-Yahr stage	3	3	3	3	3	3	3 ± 0	/
	LEDD (mg)	550/550	1300/1300	700/700	1088/1088	1850/1850	800/800	1048 ± 477	/
	MDS-UPDRS III score	29/38	40/42	37/38	50/46	45/30	55/42	43 ± 9/39 ± 5	9%
	Lateral trunk flexion (°)	5/3	12/9	25/10	20/5	5/3	5/0	12 ± 9/5 ± 4*	58%
	Anterior thoracolumbar flexion (°)	10/8	20/18	55/30	40/25	18/17	20/13	27 ± 17/19 ± 8*	32%

SCS: Spinal Cord Stimulation, LEDD: L-dopa equivalent daily dose, MDS-UPDRS III: Movement Disorder Society Unified Parkinson’s Disease Rating Scale part III

* *p* < 0.05, paired Wilcoxon’s test

All the above assessments were recorded on patients’ medication-on status after regular medications with SCS on-state

**Table 2:** Baseline clinical measurements at medication-on and medication-off state

Patient ID	1	2	3	4	5	6
Medication-on/off state						
MDS-UPDRS III	29/33	40/47	37/49	50/57	45/55	55/61
Lateral trunk flexion (°)	5/5	12/12	25/26	20/20	5/6	5/5
Anterior thoracolumbar flexion (°)	10/10	20/20	55/61	40/46	18/22	20/21

MDS-UPDRS III: Movement Disorder Society Unified Parkinson’s Disease Rating Scale part III

Three of the six patients were tested separately with SCS on-state and off-state to confirm the efficacy of SCS. All tests were performed at “medication-on” status. The results are shown in Table 3. Specific items of MDS-UPDRS III scores are presented in the Supplementary Table 2. The MDS-UPDRS III scores of all three patients were lower in the SCS on-state compared to the SCS off-state. The sagittal and coronal angles of Patient 1 and 2 improved in the SCS on-state compared to the SCS off-state. The sagittal angle of Patient 3 also improved in the SCS on-state compared to the SCS off-state. No significant improvement was observed in these patients in MDS-UPDRS part one or two, SCOPA-AUT, PDQ-39 or WCS (*p* > 0.05) (Table 4).

**Table 3:** Clinical measurements in SCS on-state and SCS off-state

Clinical Measurements (SCS on-state/SCS off-state)	Patient 1	Patient 2	Patient 3
Test time point	6 months	6 months	12 months
MDS-UPDRS III score	25/26	46/51	38/49
Lateral trunk flexion (°)	3/5	5/6	10/10
Anterior thoracolumbar flexion (°)	8/10	10/13	30/40

SCS: Spinal Cord Stimulation, MDS-UPDRS III: Movement Disorder Society Unified Parkinson’s Disease Rating Scale part III

**Table 4:** Secondary clinical measurements at pre-SCS and after SCS implantation

Patient ID (Pre-SCS/ 1-Year after SCS implantation)	1	2	3	Mean ± SD
MDS-UPDRS II	10/13	11/17	24/18	15 ± 8/16 ± 3
SCOPA-AUT	25/29	12/14	12/13	16 ± 8/19 ± 9
WCS	16/15	6/1	9/11	10 ± 5/9 ± 7
PDQ-39	23/7	31/43	70/59	41 ± 25/36 ± 27

MDS-UPDRS II: Movement Disorder Society Unified Parkinson’s Disease Rating Scale part II, SCOPA-AUT: Scale for Outcomes in PD-Autonomic, PDQ-39: 39-item quality of life questionnaire for Parkinson’s disease, WCS: Wexner constipation score

No adverse events were reported.

## Discussion

To the best of our knowledge, there has been no study about SCS application with on- and off-states in PD patients with PA. In the present study, we performed SCS surgery on the lower thoracic spinal segments of six PD patients with PA. Patients completed evaluations and were followed for 12 months, and three of them were tested separately with SCS on-state and off-state. We found that SCS improved lateral trunk flexion degree and anterior thoracolumbar flexion degree, and controlled motor symptoms without increasing the intake of anti-parkinsonism medication.

The mechanism of how SCS works on posture is not very clear. SCS may differently affect two mechanisms of postural control – reactive and anticipatory. Also SCS seems to influence cortical motor circuits involving the supplementary motor area rather than neuronal circuitries involving the brainstem and spinal cord [23]. Simultaneously, our previous study has found decreased structure/functional connectivity between the supplementary motor area and insula in PD patients with PA compared with PD patients without PA [10]. And some studies found that there was modulation of activity in primary and secondary somatosensory cortices and insula during SCS [24, 25]. Therefore, supplementary motor area and insula could play important roles in mechanism of SCS improving PA in PD.

The application of SCS to PD motor symptoms has been studied with great interest for its potential value in treating motor symptoms and gait disturbance in PD. However, previous SCS trials showed contradictory results. Some clinical trials reported negative outcomes of cervical or thoracic SCS for treating PD [6, 26], while some other studies reported that thoracic SCS resulted in significant improvements in motor symptoms and gait dysfunction [5, 7, 8, 27]. The contradiction may be explained by the variety of inclusion criteria, stimulating sites and parameters, hardware and observing outcomes. We used similar protocols and settings of SCS as previous studies [5, 28]. In our study, according to sub-scores of MDS-UPDRS III, the motor function of lower extremities was improved in most patients. This finding was consistent with some previous studies [9, 24]. Some evidence indicated that therapeutic effect of SCS might be associated with spinal segment of stimulation, though no certain pattern was found [29, 30]. In the longitudinal follow-up and comparison of SCS on/off-state, we objectively recorded a positive clinical response in postural measurements. Notably, after SCS surgery, patients’ motor function was maintained without increasing the intake of anti-parkinsonism medication, though a fluctuation in the effects of SCS on motor symptoms was observed during the one-year follow-up. Our results indicated that lower thoracic SCS had potential for treating advanced PD patients with PA. Further studies with larger sample sizes and a double-blind design are needed to warrant this conclusion.

In this study, all patients were designed to subjected to a test phase less than four weeks prior to the formal implantation of the SCS electrodes. This phase allowed both the clinicians and the patients themselves to evaluate the clinical efficacy of SCS and the patient’s tolerance to SCS stimulation. A total of seven patients were included in this trial, one of whom withdrew from the study due to unsatisfactory clinical improvements during the test phase. The remaining six patients all experienced varying degrees of clinical improvement during the test phase. These improvements included a reduction in trunk posture inclination, relief of trunk and limb rigidity, or an increase in walking speed. However, in this study, some of the clinical improvements observed during the test phase were not sustained long-term post-SCS surgery. No adverse reactions were reported by any of the patients. The short-term and long-term effects of SCS on patients with PD constitute a highly valuable research topic. Clinical trials with a large number of participants are needed.

Though SCS improved lateral trunk flexion degree and anterior thoracolumbar flexion degree in the present study, no significant improvements were observed in terms of MDS-UPDRS II (Mean ± SD, pre-surgery vs. post-surgery: 15 ± 8 vs. 16 ± 3), which was consistent with a previous study [6]. Though we found some improvements in MDS-UPDRS Q 2.9 - Turning in bed, Q 2.11 - Getting out of bed, Q 2.12 - Walking and balance and Q 2.13 – Freezing in two patients, the total score of MDS-UPDRS II was not reduced in each patient. In addition, some previous studies reported that SCS may positively impact PDQ-39 scores [11, 27]. However, in our study, while a trend towards improvement was noted, no significant improvement was found in terms of PDQ-39 (Mean ± SD, pre-surgery vs. post-surgery: 41 ± 25 vs. 36 ± 21), which was consistent with a previous study [6]. One reason of no significant improvement in MDS-UPDRS II and PDQ-39 may be the small sample size of recruited patients. Simultaneously, the progression and heterogeneity of disease may be the other reasons. Besides, the improvement of clinical measurements is not always paralleled with health status and disease progression. Autonomic dysfunction is common in PD patients and some studies [31–33] have suggested that SCS could improve cardiac autonomic function, temperature regulation and constipation. Therefore, we tried to explore whether SCS could alleviate autonomic function in PD patients, which Scale for Outcomes in PD-Autonomic (SCOPA-AUT) and Wexner constipation score (WCS) before and after SCS in three recruited PD patients were observed. However, no significant improvements were observed.

Our study has some limitations. Firstly, the small sample size limited further statistical analysis. Secondly, the assessments were not blinded, as blinding in SCS trials is challenging. This is because patients can discern not only the occurrence of paresthesia but also the variations in frequencies and voltages. Thirdly, as some participants reported exacerbated symptoms after the SCS was turned off, including fatigue, soreness in the lower limbs and back, and other discomforts. In accordance with ethical standards and the principle of no damage, we did not enforce off-state evaluations for all patients. Meanwhile, four patients missed their six-month evaluations during COVID-19 period unfortunately. Among all six patients, we did not deliberately select who would participate in the off-state evaluations, but rather based on the patients’ own willingness and tolerance to the SCS being turned off. Ultimately, three patients participated in the evaluation of the SCS off state.

## Conclusion

In conclusions, lower thoracic SCS may be effective for improving PA in PD patients. SCS has shown great promise as a supplementary therapy for PD patients with PA.

### Electronic supplementary material

Below is the link to the electronic supplementary material.

Supplementary Material 1

Supplementary Material 2

## Data Availability

All data included in this study will be shared by request from any qualified investigator.

## Abbreviations

PA: Postural abnormalities
PD: Parkinson’s disease
SCS: Spinal cord stimulation
MDS-UPDRS: Movement Disorder Society Unified Parkinson’s Disease Rating Scale
SCOPA-AUT: Scale for Outcomes in PD-Autonomic
PDQ-39: 39-item quality of life questionnaire for Parkinson’s disease
WCS: Wexner constipation score
