# Supplementary material for: Spinal cord stimulation for postural abnormalities in Parkinson’s disease: 1-year prospective pilot study
Source: BMC Neurol. 2024 May 21;24:167. doi: 10.1186/s12883-024-03673-5 (PMC11106910; doi:10.1186/s12883-024-03673-5)
Supplement: Supplementary file 2 — Supplementary Material 2 [file 12883_2024_3673_MOESM2_ESM.docx]

**Supplementary Table 1. Scores of specific items in MDS-UPDRS III for the participants before SCS surgery and at the one-year follow-up.**

| Patient ID  (pre-SCS/1-year follow-up) | 1 | 2 | 3 | 4 | 5 | 6 |
| --- | --- | --- | --- | --- | --- | --- |
| 3.1 Speech | 1/1 | 1/1 | 2/1 | 0/0 | 0/2 | 0/1 |
| 3.2 Facial expression | 1/2 | 1/2 | 3/2 | 4/4 | 2/2 | 2/2 |
| 3.3 Rigidity |  |  |  |  |  |  |
| Neck | 0/2 | 1/2 | 2/0 | 3/3 | 1/2 | 3/2 |
| Right upper extremity | 0/2 | 1/2 | 2/0 | 1/1 | 0/0 | 3/2 |
| Left upper extremity | 1/2 | 2/2 | 1/0 | 1/1 | 1/0 | 3/3 |
| Right lower extremity | 0/2 | 2/2 | 2/0 | 4/3 | 2/0 | 3/0 |
| Left lower extremity | 1/2 | 2/2 | 1/0 | 4/3 | 3/1 | 3/1 |
| 3.4 Finger tapping |  |  |  |  |  |  |
| Right hand | 0/2 | 1/1 | 2/3 | 2/1 | 2/1 | 1/0 |
| Left hand | 1/2 | 2/1 | 1/2 | 2/1 | 2/0 | 3/1 |
| 3.5 Hand movements |  |  |  |  |  |  |
| Right | 0/2 | 1/1 | 2/2 | 1/0 | 2/1 | 1/1 |
| Left | 1/1 | 2/2 | 1/2 | 0/0 | 2/2 | 2/2 |
| 3.6 Pronation-supination movements of hands |  |  |  |  |  |  |
| Right | 0/2 | 1/2 | 2/3 | 1/2 | 2/2 | 3/2 |
| Left | 1/2 | 2/2 | 1/2 | 1/2 | 3/1 | 2/2 |
| 3.7 Toe tapping |  |  |  |  |  |  |
| Right | 0/2 | 2/2 | 1/3 | 2/2 | 2/1 | 2/2 |
| Left | 1/2 | 2/1 | 1/3 | 3/2 | 3/0 | 3/2 |
| 3.8 Leg agility |  |  |  |  |  |  |
| Right | 0/2 | 2/2 | 1/2 | 3/3 | 2/1 | 2/1 |
| Left | 1/2 | 3/1 | 1/2 | 3/3 | 3/1 | 3/2 |
| 3.9 Arising from chair | 0/1 | 1/1 | 2/1 | 2/2 | 1/0 | 1/1 |
| 3.10 Gait | 2/1 | 2/2 | 1/2 | 2/2 | 2/2 | 2/4 |
| 3.11 Freezing of gait | 1/0 | 2/4 | 1/0 | 1/1 | 4/1 | 4/4 |
| 3.12 Postural stability | 0/0 | 3/3 | 2/1 | 3/3 | 2/3 | 3/3 |
| 3.13 Posture | 3/2 | 2/3 | 3/4 | 4/4 | 2/2 | 2/1 |
| 3.14 Global spontaneity of movement | 1/2 | 1/1 | 2/3 | 3/3 | 2/1 | 3/2 |
| 3.15 Postural tremor of the hands |  |  |  |  |  |  |
| Right | 0/0 | 0/0 | 0/0 | 0/0 | 0/1 | 0/0 |
| Left | 1/0 | 1/0 | 0/0 | 0/0 | 0/0 | 0/0 |
| 3.16 Kinetic tremor of the hands |  |  |  |  |  |  |
| Right | 0/0 | 0/0 | 0/0 | 0/0 | 0/0 | 0/0 |
| Left | 1/0 | 0/0 | 0/0 | 0/0 | 0/1 | 1/1 |
| 3.17 Rest tremor amplitude |  |  |  |  |  |  |
| Right upper extremity | 0/0 | 0/0 | 0/0 | 0/0 | 0/2 | 0/0 |
| Left upper extremity | 0/0 | 0/0 | 0/0 | 0/0 | 0/0 | 0/0 |
| Right lower extremity | 0/0 | 0/0 | 0/0 | 0/0 | 0/0 | 0/0 |
| Left lower extremity | 0/0 | 0/0 | 0/0 | 0/0 | 0/0 | 0/0 |
| Lip/Jaw | 0/0 | 0/0 | 0/0 | 0/0 | 0/0 | 0/0 |
| 3.18 Constancy of rest tremor | 1/0 | 0/0 | 0/0 | 0/0 | 0/0 | 0/0 |
| Total score | 29/38 | 40/42 | 37/38 | 50/46 | 45/30 | 55/42 |

**Supplementary Table 2. Scores of specific item in MDS-UPDRS III for patients with SCS on-state/off-state.**

| **Patient ID**  **(SCS on-state/SCS off-state)** | **1** | **2** | **3** |
| --- | --- | --- | --- |
| 3.1 Speech | 1/1 | 1/1 | 1/1 |
| 3.2 Facial expression | 1/1 | 1/2 | 2/2 |
| 3.3 Rigidity |  |  |  |
| Neck | 0/2 | 2/3 | 0/3 |
| Right upper extremity | 1/1 | 1/2 | 0/3 |
| Left upper extremity | 2/2 | 2/2 | 0/2 |
| Right lower extremity | 1/1 | 2/2 | 0/2 |
| Left lower extremity | 2/2 | 2/2 | 0/2 |
| 3.4 Finger tapping |  |  |  |
| Right hand | 1/1 | 2/2 | 3/3 |
| Left hand | 1/1 | 2/2 | 2/2 |
| 3.5 Hand movements |  |  |  |
| Right | 1/1 | 2/2 | 2/3 |
| Left | 1/1 | 2/2 | 2/2 |
| 3.6 Pronation-supination movements of hands |  |  |  |
| Right | 1/1 | 2/2 | 3/3 |
| Left | 1/1 | 2/2 | 2/3 |
| 3.7 Toe tapping |  |  |  |
| Right | 2/1 | 2/2 | 3/2 |
| Left | 1/1 | 2/2 | 3/2 |
| 3.8 Leg agility |  |  |  |
| Right | 1/1 | 2/2 | 2/2 |
| Left | 1/1 | 2/2 | 2/2 |
| 3.9 Arising from chair | 1/1 | 1/1 | 1/1 |
| 3.10 Gait | 1/1 | 3/3 | 2/2 |
| 3.11 Freezing of gait | 0/0 | 4/4 | 0/0 |
| 3.12 Postural stability | 0/0 | 4/4 | 1/0 |
| 3.13 Posture | 0/3 | 1/2 | 4/4 |
| 3.14 Global spontaneity of movement | 1/1 | 2/3 | 3/3 |
| 3.15 Postural tremor of the hands |  |  |  |
| Right | 0/0 | 0/0 | 0/0 |
| Left | 0/0 | 0/0 | 0/0 |
| 3.16 Kinetic tremor of the hands |  |  |  |
| Right | 0/0 | 0/0 | 0/0 |
| Left | 0/0 | 0/0 | 0/0 |
| 3.17 Rest tremor amplitude |  |  |  |
| Right upper extremity | 0/0 | 0/0 | 0/0 |
| Left upper extremity | 1/0 | 0/0 | 0/0 |
| Right lower extremity | 0/0 | 0/0 | 0/0 |
| Left lower extremity | 1/0 | 0/0 | 0/0 |
| Lip/Jaw | 0/0 | 0/0 | 0/0 |
| 3.18 Constancy of rest tremor | 1/0 | 0/0 | 0/0 |
| Total score | 25/26 | 46/51 | 38/49 |
